# English on cigarette packs from six non-Anglophone low- and middle-income countries

**DOI:** 10.1007/s00038-018-1164-9

**Published:** 2018-10-09

**Authors:** Katherine Clegg Smith, K. Welding, C. Kleb, C. Washington, J. Cohen

**Affiliations:** 1 Department of Health, Behavior and Society, Johns Hopkins Bloomberg School of Public Health, 624 N. Broadway, Baltimore, MD 21205 USA; 2 Institute for Global Tobacco Control, Johns Hopkins Bloomberg School of Public Health, Baltimore, MD 21205 USA

**Keywords:** Tobacco control, Qualitative research, Marketing, Text analysis, Product packaging

## Abstract

**Objectives:**

Low- and middle-income countries (LMICs) are vital to the global tobacco market. The pack is key to cigarette branding, and review of cigarette packs revealed English as a common feature. The prevalence of English and its potential branding utility is explored.

**Methods:**

Every available unique cigarette pack was purchased from diverse retailers in six LMICs where English is not the official language (Bangladesh, Brazil, China, Egypt, Ukraine, Vietnam). Packs’ front panels were coded for English on pack fronts. English penetration was quantified by country and a comparison of English use between multinational and national brands was undertaken. A qualitative analysis of symbolic and utilitarian usage of English was conducted.

**Results:**

Of 1303 unique cigarette packs analyzed, 67% (*n* = 876) included some English. English text conveyed product information and usage instruction. English was more prevalent for multinational brands. Qualitatively, English use frequently connected cigarettes with concepts of quality, style, luxury, and aspirational lifestyle.

**Conclusions:**

Restricting English use should be incorporated into plain packaging policy to protect populations from deceptive branding practices, specifically presenting cigarettes as an aspirational product.

## Introduction

In the twentieth century, the global market for cigarettes centered around the creation of demand in high-resource countries (Jha and Peto 2014). Over time, the tobacco market has become more global (Jha and Chaloupka 2000) due to loosened trade restrictions, rising incomes in many countries, and aggressive marketing by tobacco companies. Moreover, as cigarette consumption has declined in high-income countries, so the tobacco industry has prioritized low- and middle-income countries (LMICs) for market potential (Gilmore et al. 2015). The majority of the world’s smokers now live in LMICs, and as a result, the health impacts of smoking will now be primarily borne by people living in those locales (Jha and Peto 2014).

### Manufactured cigarettes as a symbolic product

An important condition for the creation or expansion of the cigarette market in LMICs is the emergence of a “consumer class” that has newly acquired discretionary spending power (Ustuner and Holt 2010). Over a century ago, Veblen (1899) developed the concept of “conspicuous consumption” to explain how goods could serve to demonstrate one’s social standing and status by signifying leisure and ability to spend resources without requiring total value or utility. Moreover, products can be branded in such a way as to specifically associate them with wealth or cultural power, thus symbolizing one’s connections with “the good life” (Üstüner and Holt 2009). In this paper, we consider the utility of including English on the fronts of cigarette packs purchased in countries where this is not the official language. We consider the potential opportunity for English to connect a cigarette brand with countries that have traditionally held power and resources. We also outline how English in cigarette branding may contribute to a smoker constructing and conveying a positive aspect of their identity through their brand choice (Hoek et al. 2016).

### Cigarette branding

To “brand” a product is to go beyond the attributes of the physical entity to construct associations and meanings for the product that have value to the consumer that transcend price and utility (Carter 2003). Ultimately, a brand is successful when it conveys positive attributes not only for the product but also for those who consume it (Carter 2003; Hafez and Ling 2005; Rudy 2005; Scheffels 2008; Cortese and Ling 2011; Doxey and Hammond 2011; Gendall et al. 2011).

The pack has become increasingly a key to cigarette marketing, and to creating and maintaining brand image (Hammond et al. 2009; Wakefield et al. 2002; Hoek et al. 2012; Institute for Global Tobacco Control 2013). The cigarette pack is on display when cigarettes are purchased, as well as each time a smoker retrieves a cigarette (Wakefield et al. 2012). A pack-a-day smoker might encounter a pack more than 7000 times a year (Hammond 2011). Branding on packs is also impactful for people besides the smoker (Hammond 2011); in many instances, the pack remains visible to the smoker as well as to those around them far longer than simply while the cigarette is being smoked. The cigarette pack has become ever more important as a conveyor of brand messaging as available marketing channels for tobacco companies are increasingly limited due to bans on tobacco product advertising, promotion, and sponsorship (Hammond et al. 2009; Moodie et al. 2014).

Cigarette packs convey brand information in numerous ways, including through imagery and text on the pack, and via aspects of the material pack itself (e.g., pack shape and opening design, inclusion of foil, texture of pack material). There is the potential opportunity for inclusion of brand information on each panel (side), within the pack, and on the stick itself (Smith et al. 2016). Branding text often includes brand name, manufacturer name, product slogan or tagline, and information about the product’s properties (e.g. number of sticks, size, flavor, filter).

As we have coded branding elements on packs collected through the TPackSS study (Smith et al. 2015), the prevalence of English on packs purchased in countries where English is not an official language emerged as a noteworthy aspect of pack design. We therefore conducted a focused text analysis of the appearance and use of English on the front panel of cigarette packs from a sample of non-Anglophone LMICs. *Our goal is to describe the extent of English on the primary marketing space on cigarette packs and to consider the possible communicative utilities for English in marketing of these cigarette brands*. The questions guiding this research are:

- How prevalent is English on the front panels of cigarette packs in non-Anglophone LMICs?
- What proportion of packs with any English writing on the front panel are entirely in English?
- Does the use of English on packs from brands manufactured by multinational corporations differ from that of national (or more localized) producers?
- What are the possible communicative utilities of English on English-only and some-English packs as part of branding?

## Methods

This analysis is part of a larger study of tobacco packaging in LMICs. In 2013, researchers traveled to 14 LMICs that represent countries with the greatest number of smokers and purchased one of every unique cigarette pack from 36 vendors in low, middle, and high socioeconomic areas of three major metropolitan cities (5 in China). Data collection methods are detailed elsewhere (Smith et al. 2015).

Data in this analysis are from a purposively selected subset of six (Bangladesh, Brazil, China, Egypt, Ukraine, Vietnam) countries that represent five of the six World Health Organization regions. These are also countries in which English is not an official language. We purchased and analyzed 1303 unique cigarette packs from these six non-Anglophone LMICs. All coding was undertaken by the Baltimore-based TPackSS research team; the inclusion of data from 6 countries meant that it was not possible to include researchers who were embedded within the various specific cultural contexts.

Each pack in the sample was first coded by two independent coders to determine prevalence of any English on packs as well as exclusive use of English. We estimated the level of intercoder reliability using percent agreement, a kappa statistic and the prevalence-adjusted and bias-adjusted kappa (PABAK) statistic. We established a total observed agreement of 97.5% (95% CI 96.6–98.3%), a kappa of 0.943 (95% CI 0.923–0.962), and a PABAK of 0.951. Relative to commonly used thresholds of reliability, these results would be considered “almost perfect” (Landis and Koch 1977).

In this analysis, we limit our focus to text on the front panel of the pack, as this is the primary space for brand messaging. Each pack front panel was systematically reviewed for presence of English, and text in other languages. Brand names were analyzed separately from other text. Words that were grouped together in a phrase (for example, “Quality American Blend”) were considered as a single data point. Text used in health-warning labels was excluded (even when on the front of the pack) as this language is determined by governmental policy and is not intended to convey brand messaging. In addition to analyses of English presence within each country, we compared English presence between multinational and more localized manufacturers.

Next, our communicative analysis consisted of an illustrative, qualitative taxonomy of utility and meaning conveyance of English on the pack front. From the initial pack review, we generated themes pertaining to the content conveyed by English on the pack, including use of English to describe color, place, sensation, historical references, quality, tradition, technology, romance, processing, flavor, size, and strength. In our analysis, themes were then matched with ideas from the literature on English utility (Kuppens 2010; Hornikx et al. 2010) and symbolic enhancement (Takashi 1990; Pennycook 1994; Piller 2001; Martin 2002; Kuppens 2010; Hornikx et al. 2010) in product marketing to construct and apply the following categories to the pack sample:

- Symbolic enhancement: Associations between English terminology and stereotypical American and/or British cultural attributes
- Standardization utility uses and extensions: English conveying product information or English phrasing that may be consistent across national contexts.

A team of two independent coders reviewed packs to classify English use for subthemes of either symbolic enhancement or utility usage when coding was compared. Where there was discordance in coding application, the coders discussed and made modifications where agreement could be reached. In instances where differences in perspective or confusion remained, a third coder was used and consensus was reached through further discussion. This aspect of process was qualitative and explicitly more interpretive than the coding for English presence and exclusive English, and no intercoder reliability measures were deemed appropriate or undertaken. In the findings section, beyond noting the extent of English on a pack and within a country’s sample, we do not quantify the thematic coding, but rather provide and discuss illustrative examples for each. Given that our goal was to elucidate rather than quantify use variation, we do not present concordance data for the coding process.

## Results

### Prevalence of English on packs

First, we analyzed the text on pack fronts that was neither brand name nor health-warning label; we labeled this text “appeal”. English was pervasive in appeal (see Table 1); of packs sampled, 67% (*n* = 876) included some English in the appeal content of the pack’s front panel. By country, English was most pervasive in Egypt where 95% of packs (*n* = 55) included English in appeal, in comparison to only 35% of Chinese packs (*n* = 157).

**Table 1:** English in text (appeal) or in brand name on the front of the pack

Country	Sample size	English appeals		English brand names		English appeals or brand names	
		*N*	%	*N*	%	*N*	%
Bangladesh	191	177	93	99	52	181	95
Brazil	130	106	82	100	77	122	94
China	453	157	35	61	13	183	40
Egypt	58	55	95	30	52	57	98
Ukraine	324	247	76	156	48	260	80
Vietnam	147	134	91	83	56	139	95
Total	1303	876	67	529	41	942	72

When considering the actual brand name, 41% (*n* = 529) of packs had an English brand name. This ranged from 77% in Brazil (*n* = 100) to 13% in China (*n* = 61). Combining these two components provided, an overall assessment of English prevalence in branding on pack fronts- ranging from 98% in Bangladesh to 40% in China.

We compared the use of English (in brand name and appeal) by brands that are international versus those that are not. Use of English was significantly more common for international brand packs. Any use of English was found on 79.9% of international packs versus 50.1% of national (or local) packs (*χ*^2^ = 128.1, *P* < 0.01) (see Table 2). For brand name, English was used in 51.1% of international packs versus 26.4% of national packs (*χ*^2^ = 80.3, *P* < 0.01) (see Table 3).

**Table 2:** Comparison of any English on pack fronts between multinational brands versus non-multinational brands

Brand origin (*n*)	Any English on pack front	Percentage
Multinational (750)	599	79.9
Not multinational (553)	227	50.1

*χ*^2^ = 128.1; *P* < 0.01

**Table 3:** Comparison of English in brand name between multinational brands versus non-multinational brands

Brand origin (*n*)	Any English on pack front	Percentage
Multinational (750)	383	51.1
Not multinational (553)	146	26.4

*χ*^2^ = 80.3; *P* < 0.01

Beyond having any English on the pack front, we also considered the extent to which English penetrated the textual communication in this space. Of packs with any English in appeal (not including brand name), between 52% (China) and 98% (Bangladesh) of such packs were only in English (see Table 4).

**Table 4:** Prominence of English on front of packs with any English

Country	Sample size	Any English		All English		
	*n*	*n*	%	*n*	% of packs with any English where text was all English	% of total
Bangladesh	191	177	93	173	98	91
Brazil	130	106	82	88	83	68
China	453	157	35	81	52	18
Egypt	58	55	95	53	96	91
Ukraine	324	247	76	226	91	70
Vietnam	147	134	91	121	90	82
Total	1303	876	67	742	85	57

### Uses of English on packs

Beyond quantifying the English on the pack sample, we illustrate the range of uses across packs using a qualitative coding framework developed from existing literature.

#### Brand names

Brands made use of common English terminology such as “Hello” and “More”, and also sometimes played with the language, such as in the case of the brand “Ei8ht”. English brand names drew clear connections to ideals and terminology associated with the USA (e.g., “Free”, “American Legend”, “President”, “Senator”) as well as to Britain and colonialism (“Parliament”, “Business Royals”, and “Vice Roy”). The use of such terms was often accompanied by imagery that referenced the US or Britain. English brand names also made clear references to iconic and potentially aspirational places in both the USA (“Hollywood”, “Florida” and “Texas 5”) and in Britain (“Bond Street”). English brand names made connections with power (e.g., “Mighty”, “Hero”, “Navy”, “War Horse”) as well as elegance, opulence and quality (e.g., “Silk Cut”, Gold Star”, “Blue Diamond”, “Ruby”, “Style”, “Glamour”). Brand names also made connections to romance (“Kiss”, “Charm” and “Sweet Dreams”).

There were also complexities in consideration of the use of English in brand names. Brand names like “Dunhill”, “Rothmans”, “Shelton” and “Marlboro” are not actual English words, but do have a British or American connotation in their construction. In addition, we saw (but excluded) occasional use of words associated with non-English speaking European countries, including “Monte Carlo”, “Capri”, “Armada”, and “Delta”. There was also an instance mixing of languages within a single brand—namely “Septwolves”.

**Fig. 1:**
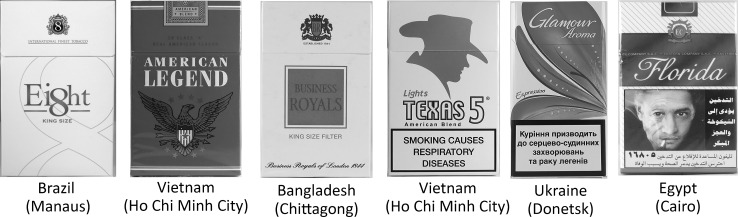
English in brand names

See Fig. 1 for example of packs from a variety of countries with English brand names.

#### Symbolic enhancement

Beyond brand names, we identified various examples of English present on pack fronts that serve to connect the product to countries with which English is associated (specifically US and England). Such connections often took the form of mentions of place, such as “Quality American Blend”, “Finest Virginia Tobacco”, “New Orleans”, “Park Avenue New York”, and “London since 1879”.

English was also frequently used to make connections between the product and concepts of quality (e.g., “perfection”, “extra quality”, “impeccable tobaccos”), style (e.g., “couture edition”, “style”, “for the stylish leader”), luxury (e.g., “black diamond”, “jet”, “silk”, “carat”), authenticity (e.g., “authentic quality”, “original”), tradition (e.g., “over 100 years of fine blending”, “100 anniversary edition”, “finest classic cigarette”, “international quality since 1872”), exceptionalism (e.g., “limited edition”, “exclusive”, “legend”), natural (e.g., “100% natural pipe tobacco”, “it will deliver exotic fresh taste by blending 20% fine cigar leaf with natural lime, mint and rum”, “green apple”, “fresh strawberry”, “bamboo”, “sequoia”, “fresh tea mix”), and wealth (e.g., “gold”, “platinum”).

English text served to connect with possible audience aspirations related to lifestyle (e.g., “fantasy”, “inspired by an original Cuban cocktail”, “vivid life of leader”, “coffee and tobacco as one”), or a memorable and positive experience (“this superbly balanced mild seven blend offers an inspiring moment of satisfaction”, “special night edition”, or “feel zesty moment”). English was also used to connote characteristics of the consumer (e.g., “innovative choice”, “elegance”, “professional”).

**Fig. 2:**
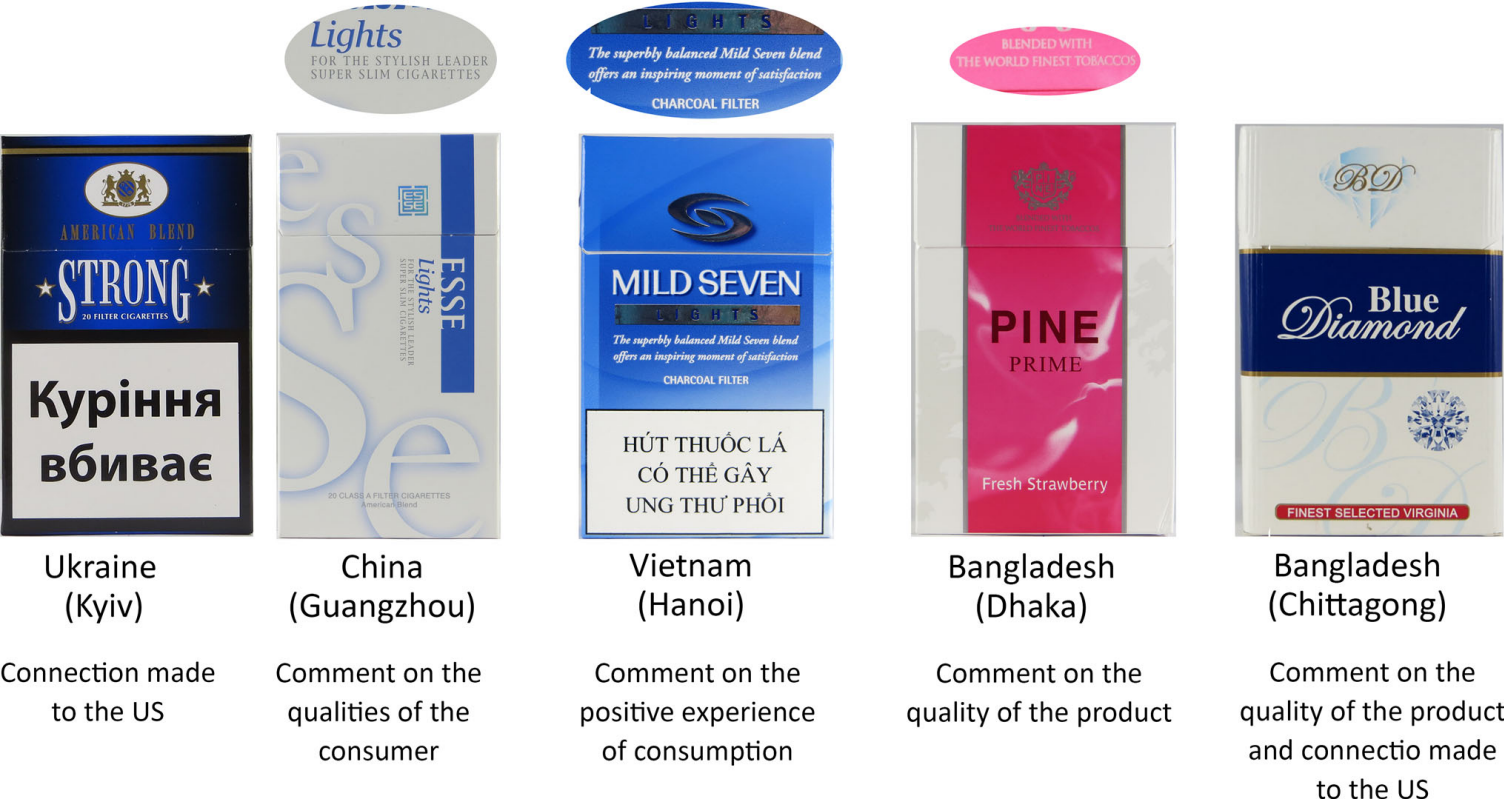
English for symbolic enhancement

See Fig. 2 for examples of packs where English possibly makes connections with aspirations for the smoker.

#### Standardization, utility uses and extensions

We also noted numerous instances of English text on the pack fronts that primarily conveyed relatively basic product information, including place of manufacture, number and size of cigarettes, type of tobacco and filter, and comparative performance characteristics (e.g., “made in Indonesia”, “20 class A cigarettes”, “charcoal filter”, “king size”, “special filter”, “less smoke smell”). English was also used (sometimes incorporating novel terminology) to convey information about how a consumer should engage with the product (e.g., “you activate fresh”, “activate 2 in 1”, “switch”), as well as expected experiences of product consumption (e.g., “totally harmony in taste”, “hd taste system”, “ice ball”), and attributes of a specific product presentation (“buy 2 packs, get a free lighter”).

By comparing packs across countries, we see that English served as part of branding consistency extending beyond national boundaries. Our coding included branding taglines or varietal descriptions that were common to multiple countries including “blend no. 555”, “classic”, “full flavor”, “master blend”, “superslims”, “super activated carbon filter”.

**Fig. 3:**
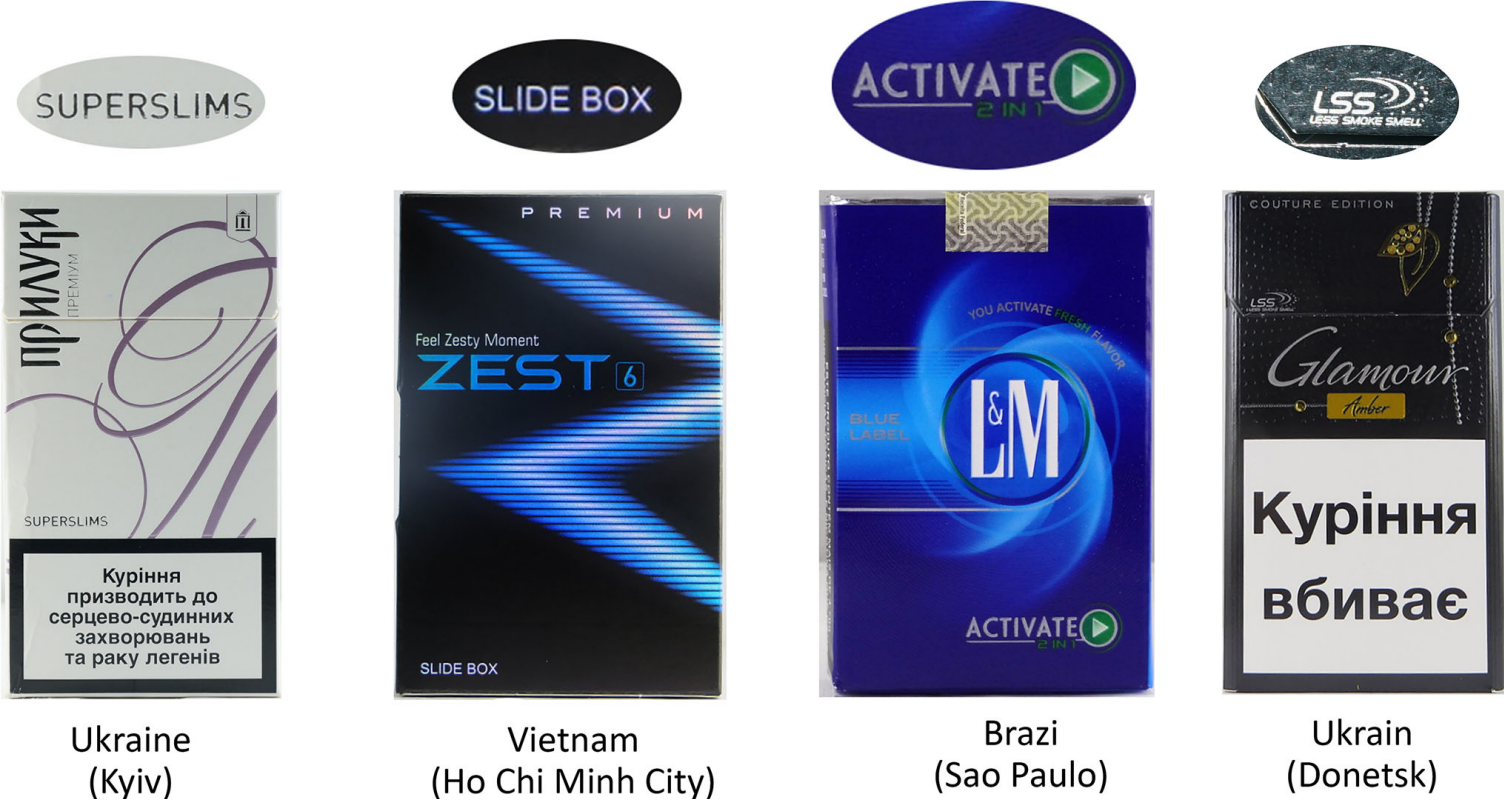
English for information or utility

See Fig. 3 for examples in which English conveyed elements of product description or instructional information about product use.

## Discussion

In our analysis, we found English to be nearly ubiquitous on cigarette packs purchased in six diverse, non-Anglophone low- and middle-income countries. English was commonly used as part of brand differentiation, both in the brand name, and in key communicative text on the primary pack face. One way to understand the widespread presence of English on the pack fronts is that this is result of the English proliferation that has been a key element in globalization as a factor in the asymmetrical flow of products, ideas and discourses that favor the perspectives of more powerful countries/cultures (Phillipson 1998; Pennycook 2017). The fact that we found significantly more English on packs from multinational companies also suggests that English may have utility in terms of homogenization and constructing a brand identity that goes across national boundaries.

We saw a norm in usage of English for basic product communication on the pack front. English terminology was used to communicate product features such as size, strength and flavor, and to convey aspirational qualities including tradition, luxury, quality, and modernity. English usage has been argued to create an economy of scale for marketing production (Hornikx et al. 2010). It is also proposed that English can sometimes fill a lexical gap; when there is no local language word to convey a concept, an English word may be used instead. Our analysis suggests that English was sometimes used for informational purposes—both in terms of the symbolic communication as well as the utility usage. Future work would benefit from a coding methodology that incorporated additional linguistic expertise in the various languages and contexts in the countries in which the packs were purchased.

We found English both by itself on pack fronts and in combination with local languages. English is often understood to be a “global language” for marketing of many consumer products (Montes 2014; Hornikx et al. 2010). Baumgardner (2006) argues that English terms can be used as “attention getters”, either on their own, or mixed with the vernacular language. Our data support the idea that including English words and phrasing on the pack is a potentially effective strategy to promote one’s brand to consumers in non-English speaking countries. It may be that there is little need for translation due to existing familiarity with the language, at least among a subset of potential consumers (Kuppens 2010).

We saw language mixing on a sizable minority of the packs on which any English was found. Prior research has found that use of English (Gerritsen et al. 2010) or use of more than one language (Martin 2002) in advertising serves to make content less comprehensible. One might conclude therefore that the use of English must have communicative value to offset possible interpretation issues generated by its inclusion. Alternatively, English may homogenize a product’s representation (Phillipson 1998), and thus potentially standardize across countries and create a global image. English may also serve as a “neutral” or somewhat familiar language in countries where more than one language is spoken (Kuppens 2010). We are not able to determine from our data which of these purposes is served by the inclusion of English, but we argue that the presence of English alongside other languages indicates that this is a deliberate communicative decision for a particular context rather than simply “spillover” of English on packs that were designed for or originated in a setting where English is the official language.

English words have a “symbolic value” in many settings (Kuppens 2010). In addition to English being potentially a channel to communicate an idea, there is also potential meaning and potential value in the act of communicating an idea in English. Our analysis demonstrates how English can contribute to the various elements of brand identity as outlined by Carter (2003). The first utility is brand as product (attributes), and we saw English being used to convey the quality of the cigarette and the tobacco contained within. Next, for brand as organization (manufacturer qualities or attributes), English was used to communicate the long and illustrative history of brand manufacturers. For brand as person (consumer attributes), we noted references to leadership, style and professionalism, and for brand as symbol (visual imagery and metaphor), we noted not only the tendency for English language brand names to be reinforced by accompanying images of iconic imagery (e.g., a landmark such as the Statue of Liberty or the Houses of Parliament, a cowboy, or an American military type crest), but also references to places that made ties to the physical product of tobacco (e.g., Virginia) as well as the lifestyle being conveyed (e.g., Hollywood, London).

This work illustrates that English on cigarette packs is a common strategy by which brand appeal is conveyed in LMICs in which English is not an official language. English is employed to convey brand value, including product strength, quality and style. English phrasing associates cigarettes with other consumable goods (such as coffee and alcoholic drinks) as well as to aspirational lifestyle qualities such as a notion of “the good life”, and connotations of relaxation and socialization. Prior research using tobacco industry documents has demonstrated how companies have sought to create brand identities that have value for populations with relatively low power (women, poor, racial minorities) in conveyance of attainment of aspirational status or lifestyles (Anderson, Glantz and Ling 2005; Brown-Johnson et al. 2014; Iglesias-Rios and Parascandola 2013). Future work might consider the extent to which English will continue to convey positive connotations in the era of Donald Trump and the possible ascendency of global powers (specifically China) for which English is not a cornerstone.

In many global settings, English continues to carry with it strong cultural connotations of youthfulness, internationalism, modernity, globalism, prestige, quality, and sophistication (Krishna and Ahluwalia 2008; Hornikx et al. 2010; Kuppens 2010). While it is true that the nature of this influence is changing in the face of interactive media platforms, English remains a powerful tool for operating in the global market place (Piller and Cho 2013; Pennycook 2017). It is not always even necessary for words to be understood to convey meaning and value for the product based upon stereotypes of the country/countries with which it is associated (Kuppens 2010; Hornikx et al. 2010). The power of English in product marketing is not limited to the message conveyed about the product itself. English can also communicate a powerful message about the consumer; one’s ability to engage with English can serve as an “ego enhancement” on the basis of the conveyance of a sense of agency and linguistic superiority (Kuppens 2010; Montes 2014). Engagement with English as part of cigarette branding may trigger aspirational sentiments, with one’s ability to comprehend and engage with the language conveying educational attainment and elevated social standing (Baumgardner 2006).

As with all studies, there are strengths and limitations associated with this work. The analysis fills an important gap in studies of marketing of tobacco products in LMICs, specifically brand marketing on the pack (Hoek et al. 2012; Moodie et al. 2014). Data collection occurred in a range of geographically and culturally diverse countries, and data collection was systematic, extensive and intended to produce as comprehensive a sample of distinct packs available for purchase as possible. As a result, we can consider both the prevalence and nature of English on cigarette packs in these non-Anglophone settings in a meaningful way. At the same time, our analysis is limited to only packs’ front panels, and we may have missed additional uses of English elsewhere. We provide a consideration of the meaning of the English words and phrases on the pack in product branding, but we acknowledge that alternative meanings are possible, and that meaning is not usually as straightforward as a single concept to a single word. We also cannot say anything about the meaning of English to the consumers as this work does not include message testing. We do not incorporate data on market share for these brands, as this is not available for all brands across all settings. Finally, our coding team was US based, and therefore, we may have missed culturally specific interpretations of English language overall, or specific terms or phrases. Future work should seek to incorporate analysts from the cultural context from which data were drawn.

There is a need for critical consideration of branding on cigarette packs in a policy environment in which opportunities to build brand identity are increasingly limited, and thus the pack is ever more important to building and maintaining a robust market. This analysis stems from the fact that the prevalence of English on our broad sample of packs from a variety of non-Anglophone LMICs was strikingly apparent from our initial review of branding elements. In our further quantitative and qualitative consideration of the packs (and the English on them), we were able to establish that English was commonly included on packs in all six countries, with China being the only country with only a minority (40%) of packs having English present on the branding space available on the front panel. English was prominent in brand names, and was included in descriptive language about product and consumer attributes. English conveys instructional information about the product and its appropriate use. In a policy context in which the pack itself is becoming more regulated (moving toward plain and standardized packaging), we argue that it is important to consider meaning conveyance in any remaining text on the pack (including brand name), as this may be an important channel by which companies seek to establish and maintain cigarettes as a status and identity-affiliated product.
